# A tumor specific antibody to aid breast cancer screening in women with dense breast tissue

**DOI:** 10.18632/genesandcancer.134

**Published:** 2017-03

**Authors:** Lopamudra Das Roy, Lloye M. Dillon, Ru Zhou, Laura J. Moore, Chad Livasy, Joe M. El-Khoury, Rahul Puri, Pinku Mukherjee

**Affiliations:** ^1^ OncoTAb, Inc., Charlotte, NC, USA; ^2^ University of North Carolina at Charlotte, Charlotte, NC, USA; ^3^ Carolinas Pathology Group, Carolinas Medical Center, Charlotte, NC, USA; ^4^ University of North Carolina at Chapel Hill, Charlotte, NC, USA; ^5^ Yale University, New Haven, CT, USA

**Keywords:** tMUC1, Breast Cancer, Dense Breast, TAB 004 antibody

## Abstract

Screening for breast cancer has predominantly been done using mammography. Unfortunately, mammograms miss 50% cancers in women with dense breast tissue. Multi-modal screenings offer the best chance of enhancing breast cancer screening effectiveness. We evaluated the use of TAB004, an antibody that recognizes the tumor form of the glycoprotein MUC1 (tMUC1), to aid early detection of breast cancer. Our experimental approach was to follow tMUC1 from the tissue into circulation. We found that 95% of human breast cancer tissues across all subtypes stained positive for TAB004. In breast cancer cell lines, we showed that the amount of tMUC1 released from tumor cells is proportional to the cell's tMUC1 expression level. Finally, we showed that TAB004 can be used to assess circulating tMUC1 levels, which when monitored in the context of cancer immunoediting, can aid earlier diagnosis of breast cancer regardless of breast tissue density. In a blinded pilot study with banked serial samples, tMUC1 levels increased significantly up to 2 years before diagnosis. Inclusion of tMUC1 monitoring as part of a multi-modal screening strategy may lead to earlier stage diagnosis of women whose cancers are missed by mammography.

## INTRODUCTION

Breast cancer (BC) is the most commonly diagnosed cancer and the leading cause of cancer-related deaths among women worldwide [1]. In 2016, the American Cancer Society estimated 246,660 new cases of invasive BC and about 40,450 deaths in the US. Worldwide this number is 1.7MM new cases and >500,000 deaths (http://globocan.iarc.fr/Default.aspx). Early detection of BC with regular screening increases the chances of survival. Screening for BC is predominantly done by mammography and clinical breast exams. While mammograms have resulted in early diagnosis for many women, 42-50% of cancers are missed in women with dense breasts due to lesion obscuration [2–7]. Increased breast density is also an independent risk factor for BC.

Given these challenges, multi-modal screenings offer the best chance of enhancing BC screening effectiveness. The American College of Radiology suggests considering supplemental ultrasonography as an option in women with dense breasts [8]. Compared with mammography, ultrasound has high sensitivity to detect BC regardless of breast tissue density, however, the specificity is low resulting in high false-positive rates [4, 5, 9, 10]. Thus there is a pressing need for the development of additional non-invasive tests that can be used in conjunction with mammography.

MUC1 (Mucin1), a membrane tethered glycoprotein, is over-expressed and aberrantly glycosylated in over 90% of BC cases. Tumor-associated MUC1 (tMUC1) is a marker of an aggressive phenotype [11, 12] that is cleaved from epithelial cells and released into circulation, allowing detection in the serum [13, 14]. MUC1 antibodies, CA 15-3 and CA 27-29, have been developed and used to detect recurrent breast cancer, monitoring the treatment of patients with advanced breast cancer and as a prognostic marker [15–17]. These tumor markers have not been used for screening since they can be elevated for benign breast lesions as well as other non-cancerous conditions (Patient Guide to Tumor markers, Carolyn Vachani RN, MSN, AOCN, The Abramson Cancer Center of the University of Pennsylvania). In this manuscript, we have evaluated the use of an antibody (TAB004), that specifically recognizes tMUC1 [18], to aid the earlier diagnosis of BC in conjunction with imaging modalities. We describe the specificity of TAB004 across major BC subtypes and a strategy that leverages the concept of cancer immunoediting [19, 20] and circulating tMUC1 measurements.

## RESULTS

### TAB004 specifically detects tMUC1 expression in human breast cancer tissue from all major subtypes

**Figure 1:**
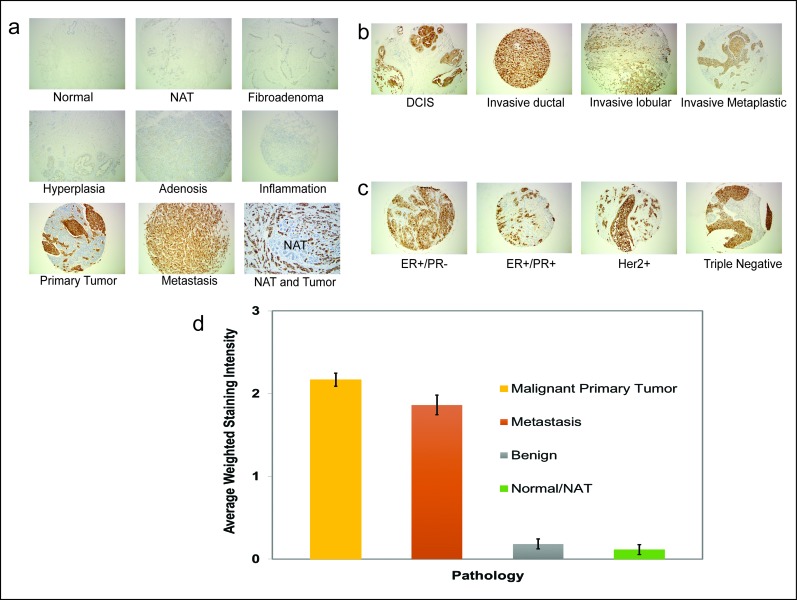
Representative immunohistochemistry images showing TAB004-HRP staining (brown) in human breast tissues **(a)** Pathology: Normal, Normal Adjacent Tissue (NAT), Fibroadenoma, Hyperplasia, Adenosis, Inflammation, Primary Tumor and Metastasis; **(b)** Carcinoma Type: Intraductal, Invasive Ductal, Invasive Lobular and Invasive Metaplastic (c) Receptor status: ER+/PR−, ER+/PR+, Her2+ and Triple Negative; and, **(d)** Average weighted staining intensity of tissue specimens by pathology. The error bars represent the standard error of the means. The images were taken at 100X magnification.

A total of 433 human BC tissue specimens were examined using immunohistochemical staining for TAB004. The tissue specimens spanned various pathology diagnoses, carcinoma type and receptor statuses (Figure 1a, 1b & 1c respectively). Primary BC and metastatic tissue demonstrated strong brown staining regardless of BC type or receptor status. In contrast the normal, benign and normal adjacent (to tumor) tissues (NAT) were mostly devoid of staining. The tissue specimens were scored on the basis of staining intensity (0 = None; 1 = Weak; 2 = Moderate; 3 = Strong) and the percent of tumor epithelial cells in the tissues positive for tMUC1 staining (0 – 100%). Based on these two measures, an average weighted staining intensity was calculated by multiplying the two scores and averaging the tissue specimens for each pathology. This quantitative comparison shows an order of magnitude stronger staining for primary BC and lymph node metastatic tissue compared to normal/NAT and benign tissue specimens (Figure 1d, p = 0.000). The data details are presented in Table 1 and shows that 95.5% of primary BC tissue specimens stained positive for TAB004 (all statistics presented in the legend).

**Table 1:** Summary of staining intensity of various tissue specimens with TAB004 **(a)** Staining intensity of normal, benign and malignant tissue specimens. Pairwise proportion test showed a statistically significant difference between normal and benign (p = 0.041) and normal/benign and malignant (p = 0.000); (tests of percent tissue stained showed no difference between carcinoma types (p-values between 0.1 and 0.738. **(b)** Staining intensity of primary tumor tissue specimens by carcinoma type. Pairwise proportion); and **(c)** Staining intensity of primary tumor specimens by receptor status. Pairwise proportion tests of percent tissue stained showed no significant difference (p-values between 0.198 and 0.640).

Table 1a: Staining Intensity of tMUC1 on human breast specimen (n=433)								
	Normal		Benign				Malignant	
	Normal (n=12)	NAT (n=72)	Fibroadenoma (N=12)	Hyperplasia (n=30)	Adenosis (n=6)	Inflammation (n=16)	Primary Tumor (n=198)	Metastasis (n=87)
Staining Intensity: 1+	2	7	3	7	4	0	24	13
Staining Intensity: ≥2+	1	0	0	2	0	0	165	65
Percent tissue specimens positive for tMUC1	25%	9.7%	25.0%	30.0%	66.7%	0.0%	95.5%	89.7%

Table 1b: Staining Intensity of tMUC1 on malignant primary tumor tissue specimens by carcinoma type (n=198)			
	Malignant primary tumors		
	Invasive Ductal Carcinoma (n=167)	Invasive Lobular Carcinoma (n=28)	Invasive Metaplastic (n=3)
Staining Intensity: 1+	19	5	0
Staining Intensity: ≥2+	142	20	3
Percent tissue specimens positive for tMUC1	96.4%	89.3%	100.0%

Table 1c: Staining Intensity of tMUC1 on malignant primary tumor tissue specimens by receptor status (n=99)			
	Receptor Status		
	ER+ and/or PR+ and/or Her2+ (n=43)	Her2+ER-PR− (n=31)	Her2+ER-PR−(TNBC) (n=25)
Staining Intensity: 1+	4	3	9
Staining Intensity: ≥2+	34	27	14
Percent tissue specimens positive for tMUC1	88.4	96.8	92.0

### tMUC1 released from tumor cells is proportional to the amount of tMUC1 present in breast cancer cells

The metabolic processes associated with MUC1 have been studied [21–23] and in addition to shedding from the cell surface, they include recycling through the Golgi apparatus and endocytosis followed by degradation. In order to understand the release of tMUC1 from cancer tissue into circulation, we studied 30 human BC cell lines with various receptor statuses (Table 2). Of these BC cell lines, 17 were derived from primary tumors and the remaining from various metastatic sites. We also included hTERT-HME1 cell line to represent normal breast cells and a positive tMUC1 control [24, 25]. Cell surface tMUC1 was assessed with flow cytometry while the total amount of tMUC1 in each cell line was measured by ELISA using cell lysate (∼15 μg/mL). The amount of tMUC1 shed from the cells was assessed by measuring tMUC1 in the cell culture media (supernatant).

**Table 2:** Receptor statuses and sites of origin for normal breast, positive control and BC cell lines studied

Cell Line	ER	PR	HER2	Primary Site	Metastatic Site
hTERT-HME1				Normal Breast	
AU-565			+	Metastatic	Pleural Effusion
HCC 202			+	Primary Tumor	
HCC 1419			+	Primary Tumor	
HCC 1954			+	Primary Tumor	
HCC 2218			+	Primary Tumor	
SK-BR-3			+	Metastatic	Pleural Effusion
UACC 893			+	Primary Tumor	
ZR-75-1	+			Metastatic	Ascites
CAMA-1	+			Metastatic	Pleural Effusion
MDA-MB-175-VII	+			Metastatic	Pleural Effusion
MDA-MB-415	+			Metastatic	Pleural Effusion
ZR-75-30	+		+	Metastatic	Ascites
MDA-MB-361	+		+	Metastatic	Brain
UACC-812	+		+	Primary Tumor	
MCF-7	+	+		Metastatic	Pleural Effusion
BT-483	+	+		Primary Tumor	
T47D	+	+		Metastatic	Pleural Effusion
HCC 38	-	-	-	Primary Tumor	
HCC 70	-	-	-	Primary Tumor	
HCC 1395	-	-	-	Primary Tumor	
HCC 1937	-	-	-	Primary Tumor	
HCC 1806	-	-	-	Primary Tumor	
DU 4475	-	-	-	Primary Tumor	
BT-549	-	-	-	Primary Tumor	
BT-20	-	-	-	Primary Tumor	
HS578T	-	-	-	Primary Tumor	
MDA-MB-157	-	-	-	Primary Tumor	
MDA-MB-231	-	-	-	Metastatic	Pleural Effusion
MDA-MB-468	-	-	-	Metastatic	Pleural Effusion
MDA-MB-453	-	-	-	Metastatic	Pericardial Effusion
KCM				Positive Control	

**Figure 2:**
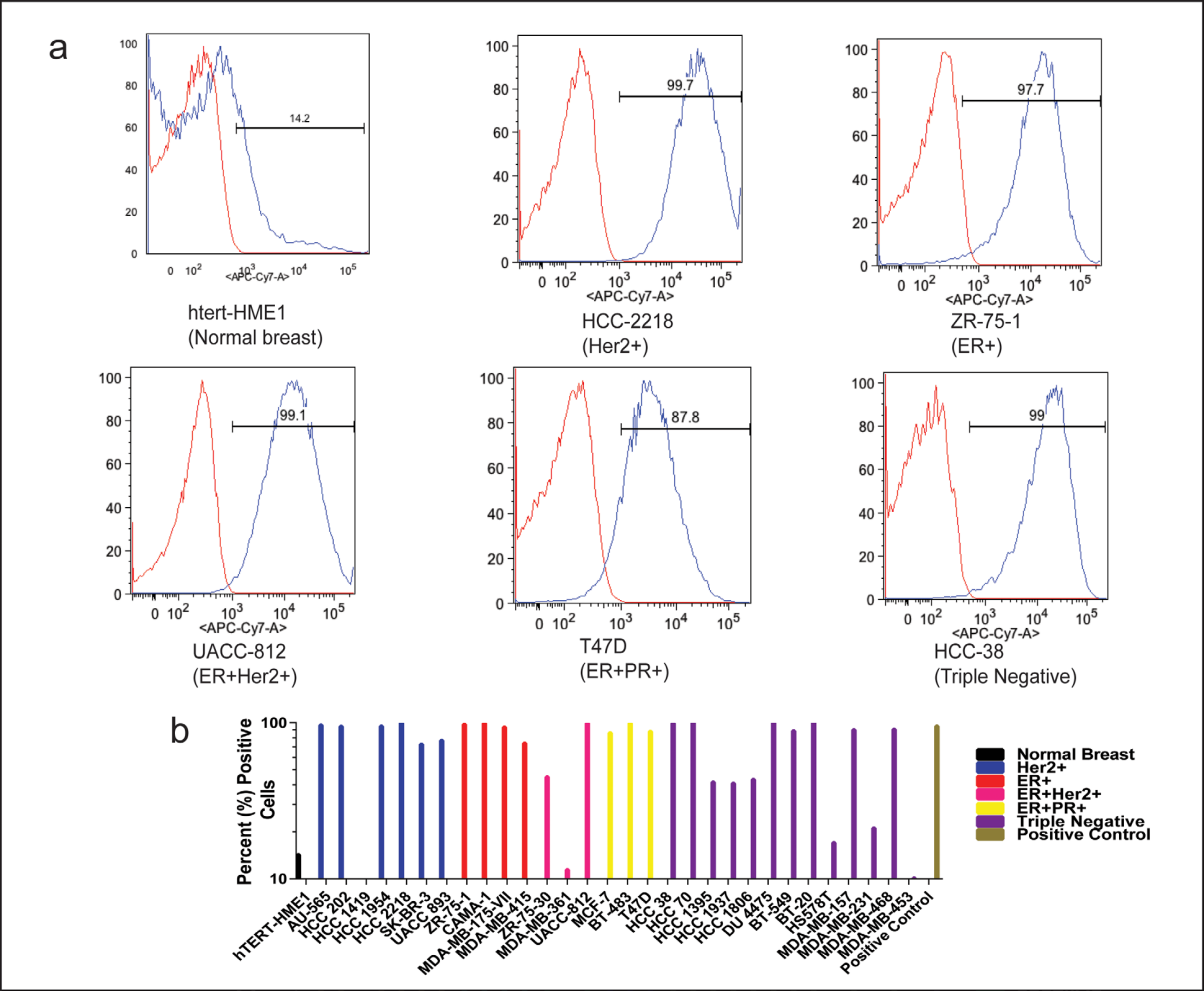
tMUC1 expression by Flow Cytometry **(a)** Representative flow cytometry analysis showing the expression of TAB004-APC-Cy7 conjugate (blue) compared to isotype-APC-Cy7 (red, negative control) in BC cell lines. **(b)** Flow cytometry results showing percent cells positive for TAB004-APC-Cy7.

Representative flow cytometry histograms from cell lines with various receptor statuses (Figure 2a) showed significant shift in fluorescence intensity from isotype control and normal breast epithelial cells, demonstrating higher expression of tMUC1 in BC cells compared to normal breast cells as detected by TAB004. The percent cells positive for TAB004-APC-Cy7 for all cell lines studied is shown in Figure 2b and represents expression of tMUC1 on the cell surface. Most of the cell line express moderate to high levels of tMUC1. Only three of the thirty cell lines (HCC1419, MDA-MB-361 & MDA-MB-453) showed lower percent cells positive for TAB004APC-Cy7 compared to hTERT-HME1. The latter two cell lines were derived from brain and pericardial effusion from the metastatic sites respectively. All the other cell lines derived from metastatic sites were from either pleural effusion (n=9) or ascites (n=2) and expressed high tMUC1. The HCC1419 cell line was developed from cells obtained from the primary tumor, however, only after chemotherapy had been administered. One other cell line studied here (UACC-812) was derived from cells following chemotherapy of a grade IV tumor and showed high percent positive cells (Source: ATCC: HCC 1419 # CRL 2326; UACC 812 # CRL 1897). We used a minimum of 1 million cells for each cell line in these experiments. The percent positive cell data reported in Figure 2b shows statistically significant difference (p = 0.000) using paired proportion tests between the various cell lines and hTERT-HME1.

**Figure 3:**
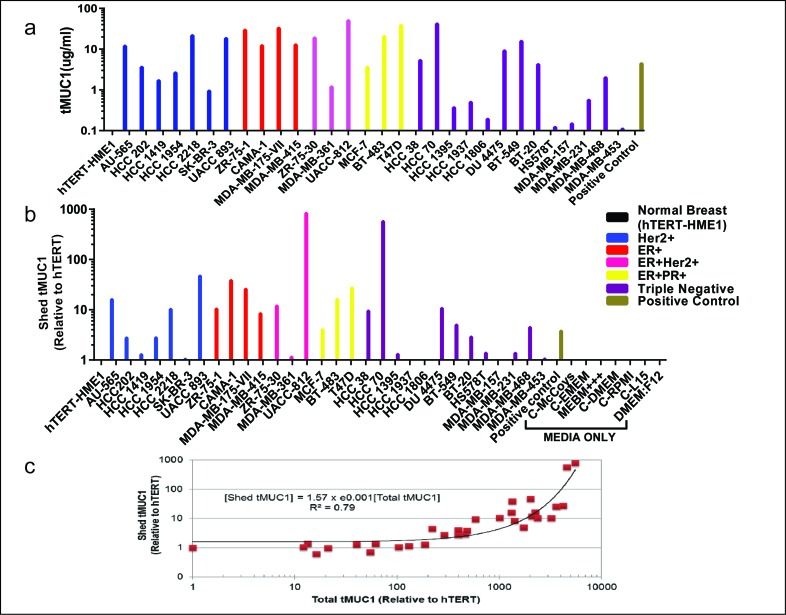
Detection of total tMUC1 and shed tMUC1 by TAB004 Elisa **(a)** ELISA results showing TAB004 detection of total tMUC1 in BC cell lines; **(b)** ELISA results showing TAB004 detection of shed tMUC1 relative to hTERT-HME1 cells; **(c)** Shed tMUC1 as a function of total tMUC1 in cancer cells.

We measured tMUC1 in total cell lysate from BC cell lines by a specific TAB004 ELISA (Figure 3a) and found that all the BC cell lines analyzed had higher total tMUC1 than the normal BC cell line hTERT-HME1 (p = .002 or lower). Interestingly, the HCC1419 and the MDA-MB-361 cell lines that showed lower percent positive cells attributable to lower surface tMUC1 expression, had higher total tMUC1 than the normal epithelial cell line suggesting higher cytoplasmic levels of tMUC1. MDA-MB-453 cells expressed low surface and total tMUC1. Western blotting was performed to confirm the expression of tMUC1 in these cell lines (Supplementary Figure 1). Cell lysates probed with TAB004 antibody revealed that a) most cell lines expressed tMUC1 regardless of the receptor status; b) hTERT-HME1 (normal breast epithelial cells) was negative for tMUC1; c) the tMUC1 levels based on densitometry analysis correlated with the tMUC1 levels determined by TAB004 ELISA (R2 of 0.96) (Supplemental Figure 2).

In addition, we determined the amount of tMUC1 shed from the BC cell lines into the media used to grow the cell lines relative to hTERT-HME1, by TAB004 ELISA (Figure 3b). Media only was included to demonstrate that there was no interference in the shed tMUC1 measurement. Nine of the thirty breast cancer cell lines studied did not show any statistically significant difference (p > .05) from the levels shed from the hTERT-HME1 cell line. In order to better understand the levels of shed tMUC1 relative to the total tMUC1 present in BC cells, we compared ELISA data from the cell supernatant to that of the cell lysate for each of the cell lines studied. We normalized the ELISA values relative to hTERT-HME1 (human normal mammary epithelium) for this comparison (Figure 3c). The levels of shed tMUC1 showed an interesting correlation to the amount of total tMUC1 in the lysate of the cells. There appears to be a threshold of total tMUC1 level (∼ 200x that of hTERT-HME1) before shed tMUC1 increases at an exponential rate. No other factor examined (site of origin, cell type and BC subtype) explained the difference in low shedding versus high shedding cell lines.

### Circulating tMUC1 levels can be detected in healthy individuals and increases with disease progression in breast cancer patients

Circulating tMUC1 levels were measured in 559 serum samples obtained from women using an ELISA that was developed with the TAB004 antibody (commercial name Agkura^™^ Personal Score). We designed a standard using protein lysate isolated from a MUC1-expressing tumor cell line (KCM), which was also used to generate the TAB004 antibody. The serum samples were collected following consent from healthy volunteers as well as from women who were diagnosed with benign breast disease or invasive BC spanning all stages. Some of these samples were serial measurements from the same women. The samples were obtained from multiple cohorts represented by: (i) Fox Chase Cancer Center (FCCC), (ii) Women and Infants Hospital of Rhode Island (WIHRI), (iii) Carolinas Healthcare System (CHS), (iv) University of North Carolina at Charlotte (UNCC), and (v) Abcodia, Inc/University College of London (Abcodia-UCL). The serum samples from Abcodia-UCL were from volunteers who participated in the UK Collaborative Trial of Ovarian Cancer Screening (UKCTOCS). Three time points leading to diagnosis were provided for 30 cases and 30 matched controls. These samples were provided with patient diagnosis blinded and was revealed only after we shared the tMUC1 values with our collaborators at Abcodia-UCL.

**Figure 4:**
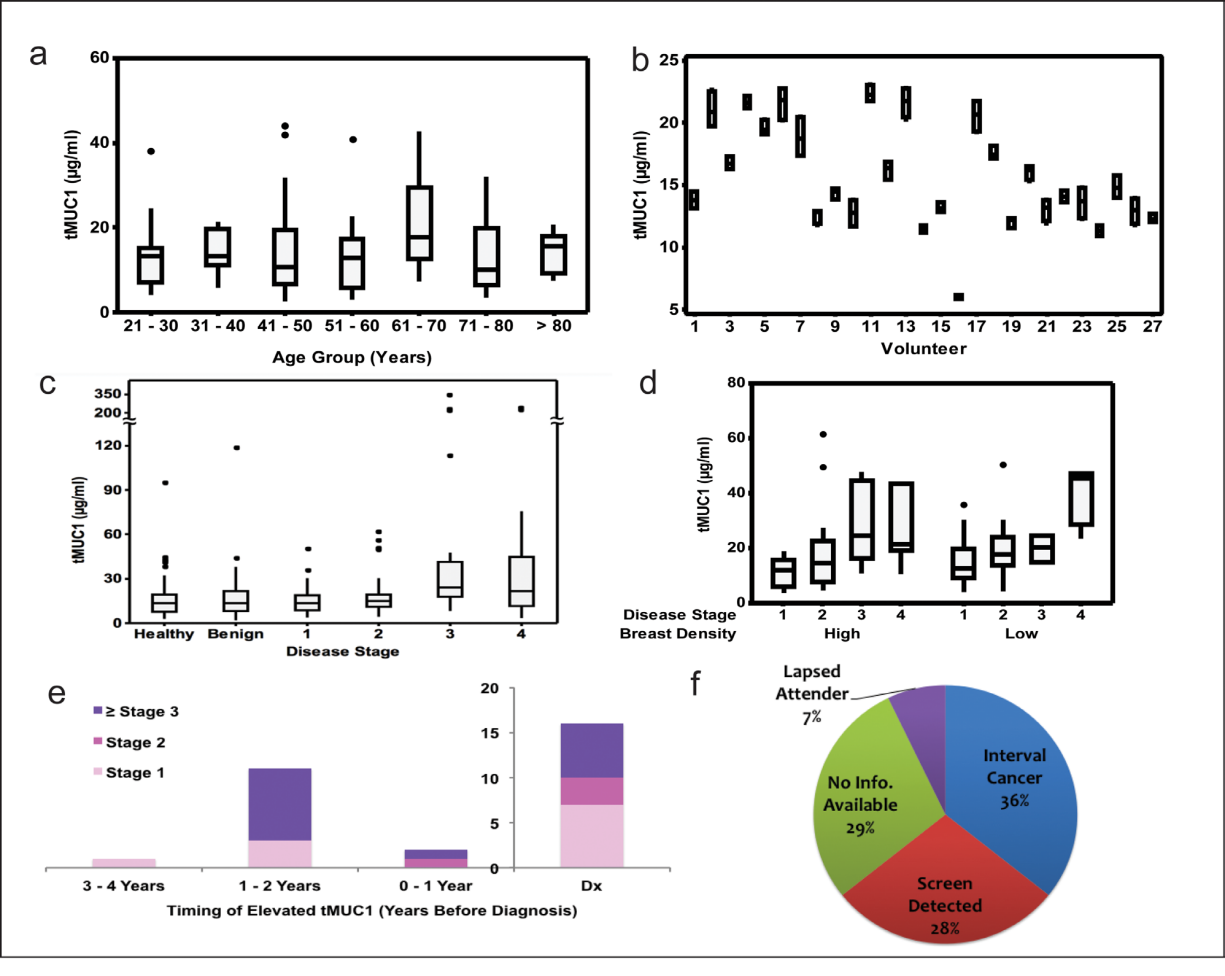
Circulating shed tMUC1 measurements in multiple cohorts **(a)** Healthy volunteers by various age groups (FCCC, CHS) (ANOVA, p=0.614), **(b)** tMUC1 measurements in healthy volunteers obtained 15 days apart (UNCC), **(c)** Healthy volunteers and women with benign breast disease or invasive BC across all stages (FCCC, WIHRI, CHS), **(d)** Invasive BC across all stages and breast density categories (FCCC, WIHRI) show no significant difference between high and low density (p = 0.102) and statistically significant difference between early and late stage disease (p = 0.006), **(e)** Timing of elevated/increasing tMUC1 measurements relative to diagnosis (Abcodia-UCL), and (f) Screening mammography status of invasive BC cases with elevated/increasing tMUC1 measurements prior to diagnosis (Abcodia-UCL). The asterisks in Figures 4a-4d represent outlier data points.

In healthy volunteers of different ages, we found no difference in the circulating tMUC1 level between women of different age groups (Figure 4a). While the measurements for the women in the age group 61-70 years visually appear to be higher, there was no statistically significant difference between the age groups (ANOVA p-value = 0.614). This data set was used to calculate the reference interval to be < 31.4 μg/ml for this TAB004 ELISA.

Biological variation was studied using healthy volunteers (n = 27: UNCC) whose samples were collected 15 days apart. The idea of collecting their samples over a relatively short duration span was to capture the variation in their tMUC1 measurement for reasons other than cancer progression in the event there are undiagnosed cancer cases in this cohort. Two measurements were taken on each day and the 4 measurements of each of the volunteers are shown as boxplots in Figure 4b. The statistical analysis to estimate components of biological variation was conducted using methods described in the literature [26]. The 95% confidence level of acceptable normal variation (measurement and biological combined) or reference change value was calculated to be 10%. We found that each volunteer had some variation around their own personal score that can be very different from the personal scores of other volunteers.

Circulating tMUC1 measurements for healthy volunteers as well as women diagnosed with benign disease or invasive cancer (n=325: FCCC, CHS and WIHRI) are shown as boxplots in Figure 4c. These results were analyzed for statistical significance using paired T-tests between all the diagnosis groups. The healthy volunteers had lower tMUC1 levels compared to Stages 2, 3 & 4 breast cancer patients (p = 0.039 or lower). The women diagnosed with benign breast disease had lower tMUC1 levels than Stages 3 & 4 breast cancer patients (p = .006 or lower). Note that this analysis is affected by a single significant outlier data point in the benign set (tMUC1 = 118 μg/ml, Figure 4c). Elimination of this data point resulted in the tMUC1 levels for the women with benign breast disease to be lower than Stages 2, 3 & 4 breast cancer patients (p = 0.036 or lower). The levels of tMUC1 in breast cancer patients could be differentiated by stage with statistical significance (p = 0.031 or lower) with Stages 3 & 4 treated as one group. There was no difference in tMUC1 levels between Stage 3 and 4 breast cancer patients. One of our key objectives was to understand if there was any effect of breast tissue density on circulating levels of tMUC1. Figure 4d presents data from a subset of this cohort (n = 100) for whom breast density information is available. The figure shows boxplots for each BC stage and across low and high breast tissue densities. Statistical analysis showed no significant difference in the tMUC1 values between high and low tissue density across all stages of the disease (t-test p-value = 0.102). Both high and low tissue density, showed an increase in the tMUC1 values as the BC stage increased (t-test p-value = 0.006 for early stages 1 and 2 versus late stages 3 and 4). This is a clinically significant finding because most women with high breast tissue density are diagnosed at stage 3 and above since mammograms repeatedly miss their cancers.

Given that tMUC1 levels vary significantly between individuals but are steady over time in healthy individuals (see Figure 4b); and increase with disease progression in breast cancer patients (see Figures 4c & 4d), we hypothesize that monitoring individuals over time and comparing to their own baseline score may detect breast cancer. To test this hypothesis, the Abcodia-UCL blinded study (n = 180 samples from 60 volunteers) was analyzed by evaluating if the tMUC1 level either (a) exceeded the reference interval of < 31.4 μg/ml, or (b) measurements increased more than the 10% reference change value threshold compared to the first time point measurement. Overall, 47% of all cases and 60% of the late stage cases triggered one of these criteria. Only one case triggered criterion (a) 3-4 years prior to detection by screening mammography. All cases triggered criterion (b), mostly 1-2 years prior to diagnosis (Figure 4e). The mammography screening status of the cases that triggered these criteria are shown in Figure 4f. A third of these cases were interval cancers confirming that they were missed by mammography. While roughly another third were detected by screening mammography. Given that a majority of the screen detected cases were late stage, it is possible these women had tumors that were not picked up on an earlier mammogram. Unfortunately, the breast density status of this cohort is not known. Nonetheless, this data supports the use of the circulating tMUC1 measurement in conjunction with mammography as an aid to earlier diagnosis of BC using the 2 criteria mentioned above.

The Abcodia-UCL cohort also included 30 controls from the UKCTOCS volunteers that did not have a cancer diagnosis. At the time of analysis, two of these controls were reported to have had cancer and have been removed from the analysis. For the remaining 28 controls, tMUC1 measurement for one volunteer triggered criteria (a) and 36% triggered criteria (b) listed above. Of the 2 controls that were removed from analysis, one met both criteria (a) and (b). This individual was diagnosed with esophageal cancer 11 years after criterion (a) was met. Given that the time elapsed between initial carcinogen exposure to clinical detection of most solid tumors spans two decades [27, 28], there is no way to be sure that the controls that triggered the criteria were not developing cancers that would be detected in the future.

Given this challenge, we compared the percent increase in tMUC1 over time of the BC cases from the Abcodia-UCL cohort with the healthy volunteers from the biological variation study. We found the increase in tMUC1 for the Abcodia-UCL cases was statistically significant compared to the healthy volunteers (t-Test, p = 0.004).

### TAB004 can target tMUC1 *in vivo* and be used to image breast cancer

**Figure 5:**
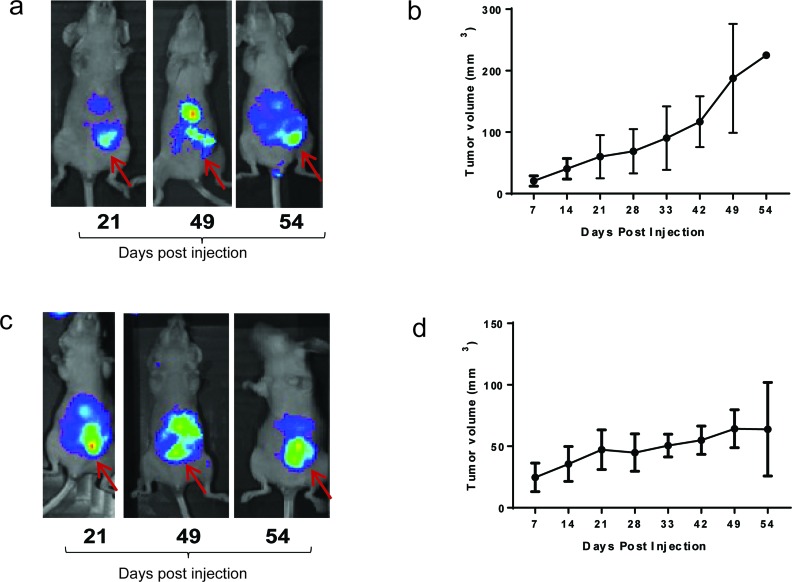
TAB004 –ICG fluorescence and tumor growth **(a)** HCC70 Orthotopic model, and **(b)** AU565 orthotopic model.

Given our findings that TAB004 recognized tMUC1 on 95% of BC tissue specimens and that a certain threshold of total tMUC1 in the cancer cells is needed before tMUC1 is released into circulation, we next investigated *in vivo* imaging of tMUC1 using TAB004 to explore its potential for imaging applications. We have successfully demonstrated *in vivo* fluorescence imaging of spontaneous BC tumors in the MMT mouse model that has the human MUC1 protein and closely mimics human BC progression using Indocyanine Green (ICG) labeled TAB004 [29]. In this study our objective was to compare images of orthotopic tumors generated in mammary fat pad with a high and a moderate tMUC1 shedding cell line with different receptor statuses. We selected the HCC70 (TNBC) and the AU565 (Her2+ type) cell lines respectively for this comparison. Our study showed HCC70 shed 36 fold higher tMUC1 compared to AU565. Images from TAB004-ICG fluorescence in these orthotopic mouse models were taken at 3 time points: 21, 49 and 54 days post injection of the cancer cells (Figure 5) with the tumor locations identified by arrows. The images show the strongest signals from the tumor locations. The background signal is due to the fact that ICG fluoresces from 750 nm to 950 nm, which overlaps with infrared thermal radiation from the mouse. Tumor volume data documenting the growth of the tumors is plotted adjacent to the images. Tumor growth is considerably slower in the AU565 orthotopic model. Despite the differences in the levels of shed tMUC1 from these cell lines, TAB004-ICG targets the tumors equitably. This is consistent with our flow cytometry data that showed comparable percent positive cells for the two cell lines.

## DISCUSSION

MUC1 has long been associated with BC [30] and has been prioritized as one of the most important cancer antigens [31]. This study has systematically examined the use of the TAB004 antibody, which specifically targets the tumor form of this glycoprotein (tMUC1), to address the late stage diagnosis of BC, particularly in women with dense breast tissue. Traditionally, *in vitro* diagnostics rely on either a single cutoff or a population based reference value to differentiate between healthy and diseased states. The usefulness of population based reference values for an analyte can be assessed by comparing the ratio of within-subject biological variation to the between-subject biological variation, or the “index of individuality”[32]. The index of individuality needs to be higher than 1 for a population based reference interval to be useful in clinical practice [32]. For tMUC1, the index of individuality is 0.11, indicating serial measurements and the use of the reference change value is more appropriate.

There is a scientific basis to the use of serial measurement and the reference change value. Over a century ago, it was first proposed that cells are continuously transformed in our bodies and the immune system eliminates these cells, a concept referred to as immune surveillance [19]. It took the scientific community more than 80 years to accept this idea which has matured to the concept of cancer immunoediting with 3 phases: elimination, equilibrium and escape [20]. In the first two phases, the immune system either eliminates the cancer cells or keeps them in check, preventing them from growing. Since the glycosylation structure of MUC1 changes during the transformation of normal to cancer cells (forming tMUC1), the elimination and equilibrium phases explain the presence of tMUC1 even in healthy individuals. The levels of tMUC1 would be expected to remain steady for each individual as long as the equilibrium phase is maintained. It is conceivable that the level of tMUC1 in individuals would differ depending on the number of transformed cells in the equilibrium phase and on the extent to which tMUC1 is shed from these transformed cells. These ideas are supported by the data (Figure 4b) showing high individuality of tMUC1 levels that are steady over time. The escape phase of cancer immunoediting is reached when immune resistant tumor cell variants develop causing tumor growth. Our data (Figure 4d) shows a significant increase in tMUC1 levels with increase in stage or tumor growth. Imaging modalities detect BC in the escape phase of cancer immunoediting [20]. The data presented in Figures 4e-4f support that serial monitoring of tMUC1 using the TAB004 antibody can lead to earlier diagnosis in conjunction with imaging. Given that TAB004 recognizes tMUC1 across all BC subtypes (Figures 1, 2, and 3), a TAB004 based ELISA can be successfully used for screening as an adjunct to mammography. An increase in the tMUC1 level would trigger the use of ultrasounds and MRI's to result in earlier diagnosis relative to mammography alone. Unfortunately, the potential false positive rate of this strategy can't be assessed based on the studies done to date and additional studies are needed.

Opportunities exist to develop TAB004 for *in vivo* use with existing imaging modalities to enhance the early detection of BC and develop targeted therapies, especially for triple negative BC, which has no target. We have recently published that nanoparticles conjugated to TAB004 specifically target the tumor site in a model of spontaneous BC (MMT mice) [33].

These findings are highly significant given that considerable resources continue to be invested in biomarker development [34, 35]. The Early Detection Research Network (EDRN) (https://edrn.nci.nih.gov/biomarkers#b_start=0&c0=Breast) lists 195 BC protein and gene biomarkers, however none of them have been adapted for BC screening using EDRN's reference sets. This maybe because the reference sets are based on single blood draws and clinical studies are striving to identify one cut-off value to discriminate between benign and malignant samples. The penchant for a single cut-off value and failure to discriminate with one biomarker has lead researchers to attempt panels of biomarkers. Considering cancer immunoediting, it is not clear if such an approach will work for BC screening.

## MATERIALS AND METHODS

Generation of antibody: TAB004, a mouse IgG1 monoclonal antibody (Patents: US 8,518,405 & 9,090,698; Japan 5,886,299; Australia 2,011,312,830 B2; China ZL201180059040.8; Russia 2595403) was generated as previously published [18] This antibody specifically recognizes an epitope on the peptide core of tumor-associated mucin1 (tMUC1). TAB004 antibody was custom manufactured by LakePharma Inc, Belmont, CA for the studies described in this manuscript.

### Immunohistochemistry

The following tissue arrays were purchased from US Biomax Inc, Rockville, MD 20855, USA: 1) # BR723: Breast various pathology developmental process, including TNM (primary tumor, lymph nodes, metastasis) and pathology grade; 2) #BR2082: Breast disease spectrum (breast cancer progression); 3) #BR10010b: Breast cancer and matched metastatic carcinoma, including TNM and pathology grade, with ER, PR and Her-2 (neu); 4) #BR801a: Breast adjacent normal tissue array with breast cancer tissue, including TNM, clinical stage and pathology grade. Tissues were deparaffinized and hydrated via washes with 100% EtOH, 95% EtOH, 70% EtOH, water and then slides transferred to 1X phosphate buffer saline (PBS). Antigen retrieval was performed for 45 min at 99°C followed by a 20-minute cool down (RT) in Dako Target antigen retrieval (S1700, Dako, Carpinteria, CA). The endogenous peroxidase activity of the sections were blocked by pretreatment of tissues with 2% Hydrogen Peroxide (Catalog # H323-500, Stock concentration: 30%, Fisher Scientific) in 100% Methanol for 10 minutes. Sections were washed with 1XPBS in between these steps. Sections were then blocked for 1 hr in 50% FBS in 1XPBS, and then incubated over-night at 40C with 100-200μl diluted TAB004-HRP [(Stock: 0.51mg/ml; Lot#878795BC; needed 1μg in 750 μl. Final concentration to be used: 1.96μl diluted in 750μl of 15%FBS/1XPBS)]. Sections were then washed 4 times with 1XPBS. For all slides, 3, 3″–Diaminobenzidine (Vector Laboratories, Burlington, CA) was used as the chromogen and hematoxylin was used as counterstain. Slides were then dehydrated, cover slipped with permount, and viewed using light microscopy. Each core on the tissue array was reviewed and scored by Dr. Chad A. Livasy, MD, a breast pathologist for the Carolinas HealthCare System (Carolinas Pathology Group, Carolinas Medical Center, Charlotte NC). The scoring included two attributes: a) the intensity of the staining, and b) the percent of epithelial cells stained. All scoring was done over two sessions to ensure consistency of scoring across cores.

### Cell culture

A comprehensive set of BC cell lines were derived from American Type Culture Collection (ATCC, Manassas, VA 20110, USA) master seed stocks to eliminate variability (ATCC breast cancer cell panel 30-4500K). We used the following cell lines for our study: hTERT-HME1# CRL-4010, AU-565 #CRL-2351, HCC 202# CRL-2316, HCC 1419# CRL 2326, HCC 1954#CRL 2338, HCC 2218# CRL 2343, SK-BR-3# HTB-30, UACC 893# CRL 1902, ZR-75-1# CRL-1500, CAMA-1# HTB-21, MDA-MB-175-VII# HTB-25, MDA-MB-415# HTB-128, ZR75-30# CRL-1504, MDA-MB-361# HTB-27, UACC-812# CRL-1897, MCF-7# HTB-22, BT483# HTB-121, T47D# HTB-133, HCC 38# CRL-2314, HCC 70# CRL-2315, HCC 1395# CRL-2324, HCC 1937# CRL-2336, HCC 1806# CRL-2335, DU 4475# HTB-123, BT-549# HTB-122, BT-20# HTB-19, HS 578T# HTB-126, MDA-MB-157# HTB-24, MDA-MB-231# HTB-26, MDA-MB-468# HTB-132, MDA-MB-453# HTB-131. Each cell line was cultured in accordance with the culture method provided by ATCC. The positive control cell line, KCM, was isolated from a pancreatic tumor in a mouse that was transgenic for human MUC1 [24, 25]. These cells were maintained in DMEM (Invitrogen-Thermo Fisher Scientific, Grand Island, NY) containing 10% FCS, 1% penicillin/streptomycin and 1% glutaMAX™.

### Western blotting

Cellular lysate preparation and Western blotting was done as previously described [36]. A 5% SDS-PAGE was used and 15μgs of cell lysate was loaded. 1:10,000 TAB 004 mouse monoclonal anti-human MUC1 antibody was used to probe for MUC1-TR (18). TAB 004 recognizes the STAPPVHNV epitope within MUC1 TR [37]. Membranes were also probed for β-actin (Santa Cruz) to account for equal loading of the protein. Rabbit anti-mouse secondary antibody was used from Santa Cruz, at 1:5000. The NIH Imaging program was used to conduct densitometry analyses of immunoblots as shown in Supplementary Figure 1b. Results are presented as mean values of arbitrary densitometry units corrected for background intensity and normalized to the expression of b-actin.

### Orthotopic mammary tumor models

#### a Ethics statement

All experimental procedures were conducted according to Institutional Animal Care and Use Committee (IACUC) guidelines and the IACUC Committee of University of North Carolina at Charlotte (UNCC) has specifically approved this study (IACUC ID: 13-009). All mice were bred and maintained in a pathogen free facility.

#### b Induction of tumors for imaging

Six – eight weeks old female, athymic nude mice purchased from Envigo Laboratories, Indianapolis, USA ((order code: 069(nu)/070(nu/+)) were injected with 5 × 10^6^ breast cancer (BC) cells ((AU565: Her2+ and HCC 70 (Triple negative)) in 100μl of 1XPBS in the mammary fat pad. Mice were palpated starting at 6 days post tumor injection. Tumor weight was calculated according to this formula: weight in grams=[length in centimeters x (width in centimeters)^2^ ]/2.

We used the IVIS *in vivo* imaging system (Perkin Elmer, Waltham, MA) to monitor tumor progression TAB004 antibody was conjugated with fluorophore Indocyanine green (TAB-ICG, Dojindo Molecular Technologies, MD, USA). Prior to imaging the mice, TAB-ICG in sterile saline was administered retro-orbitally (RO) in mice and imaging was conducted 24 hours post TAB-ICG injections. Mice bearing AU565 and HCC70 tumors were imaged at 21, 49 and 54 days post tumor inoculation.

Tumor fluorescence was analyzed using the Life Science Software Suite (Perkin Elmer) and region of interest were defined at tumor location. In parallel, the presence of tumor masses was assessed by palpation and recorded weekly.

### Flow cytometry

To assess tMUC1 expression, cells were harvested and stained with TAB004 conjugated to APCCy7 (Abcam, Cambridge, MA, USA), or stained with APC-Cy7 mouse IgG1 isotype control (BD Biosciences, San Jose, CA, USA). Data was collected using the BD LSR Fortessa Flow Cytometer (BD Biosciences) using filter for Cy5.5 (7-AAD) and Cy7 (TAB-004) and analyzed with FlowJo software (version 8.8.7; FLOWJO, Ashland, OR, USA).

### BCA protein assay

To measure tMUC1 protein levels in the total cell lysate of BC cell lines, we performed TAB004 Elisa. We loaded 15μg/ml of protein for each cell line. Total protein in BC cell lines was assessed by BCA assay (Pierce BCA protein assay kit, #23225; Thermo Fisher Scientific, Grand Island, NY) and followed manufacturer's recommendation to perform the assay.

### ELISA

The Agkura^™^ Personal Score Enzyme Immunoassay for tMUC1 (OncoTAb, Inc. Charlotte, NC) is a sandwich solid phase enzyme-linked immunosorbent assay (ELISA). The standard used is the KCM cell line lysate. During this assay, the test sample is allowed to react first with the immobilized TAB004 antibody at 25° for one hour. The wells are washed with wash buffer to remove unbound antigen. The TAB004-HRP conjugate is then added for 1 hour at 25°C allowing it to react with the immobilized antigen which results in tMUC1 being sandwiched between the solid phase and the enzyme-linked antibodies. The wells are washed to remove unbound labeled antibodies. TMB reagent, which detects horseradish peroxidase (HRP) activity, was added and incubated for 20 minutes, resulting in the formation of a blue color. This color development was stopped with the addition of Stop Solution changing the color to yellow. The concentration of tMUC1 is directly proportional to the color intensity of the test sample. Absorbance was measured spectrophotometrically at 450 nm and used to calculate tMUC1 concentration to report the Agkura^™^ Personal score.

### Statistical Analysis

Statistical analysis was done for the circulating tMUC1 data (Figure 4) and the *in vivo* data (Figure 5). Significance of the data presented in Figures 4a - 4d was determined by ANOVA and one tail t-tests. Minitab v.17 and Excel was used for these analyses respectively. No statistical method was used to determine sample size for the various diagnosis categories (healthy = 124, benign = 51, stages 1 = 53, stage 2 = 36, stage 3 = 28, stage 4 = 33). For the biological variation analysis (Figure 4b), the method described by Blackwell et al (26) was used. Data from 3 volunteers was excluded in accordance with the method since they were outliers exceeding ± 3 SD. Data from another 2 volunteers was excluded since their tMUC1 values were below the limit of detection. For Figure 5, data was analyzed using graphpad software. Results are expressed as mean ± s.e.m.

## SUPPLEMENTARY MATERIALS FIGURES



## Abbreviations

MUC1: Mucin 1
tMUC1: Tumor associated MUC1
BC: Breast Cancer
NAT: Normal adjacent (to tumor) tissue
