# Supplementary material for: A tumor specific antibody to aid breast cancer screening in women with dense breast tissue
Source: Genes Cancer. 2017 Mar;8(3-4):536–49. doi: 10.18632/genesandcancer.134 (PMC5489651; doi:10.18632/genesandcancer.134)
Supplement: Supplementary file 1 [file ganc-08-536-s001.pdf]

A tumor specific antibody to aid Breast Cancer screening in women with dense breast tissue

Lopamudra Das Roy[1,2], Lloye M. Dillon[1,2], Ru Zhou[2], Laura J. Moore[2], Chad Livasy[3,4], Joe El Khoury[5], Rahul Puri[1], Pinku Mukherjee[1,2]

Supplementary Materials

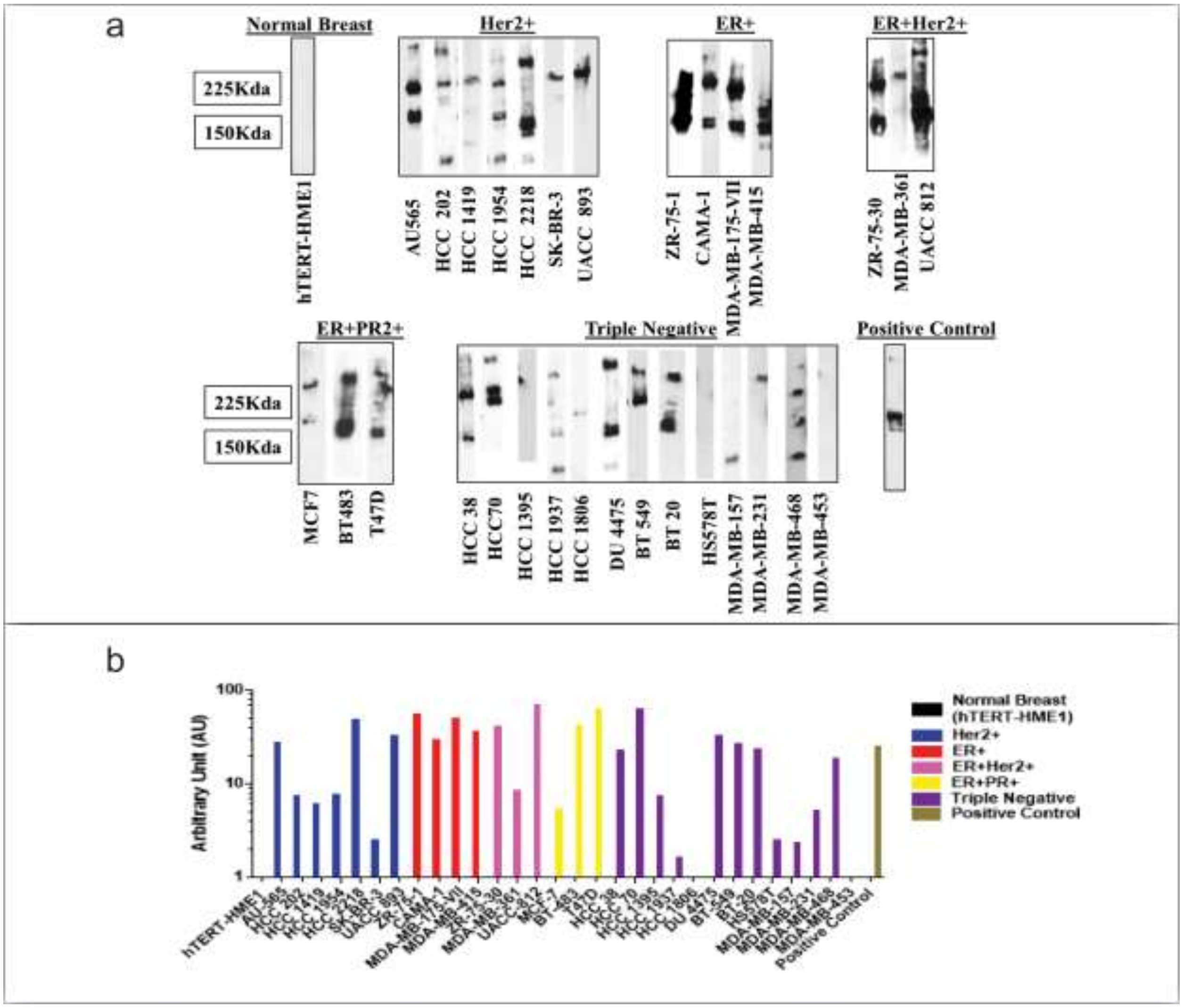

Supplementary Figure 1: tMUC1 expression by Western Blotting: (a) Western blot analysis using TAB004 to measure tMUC1 protein levels in total cell lysate from breast cancer cell lines, (b) Densitometry analysis of western blots from breast cancer cell lines

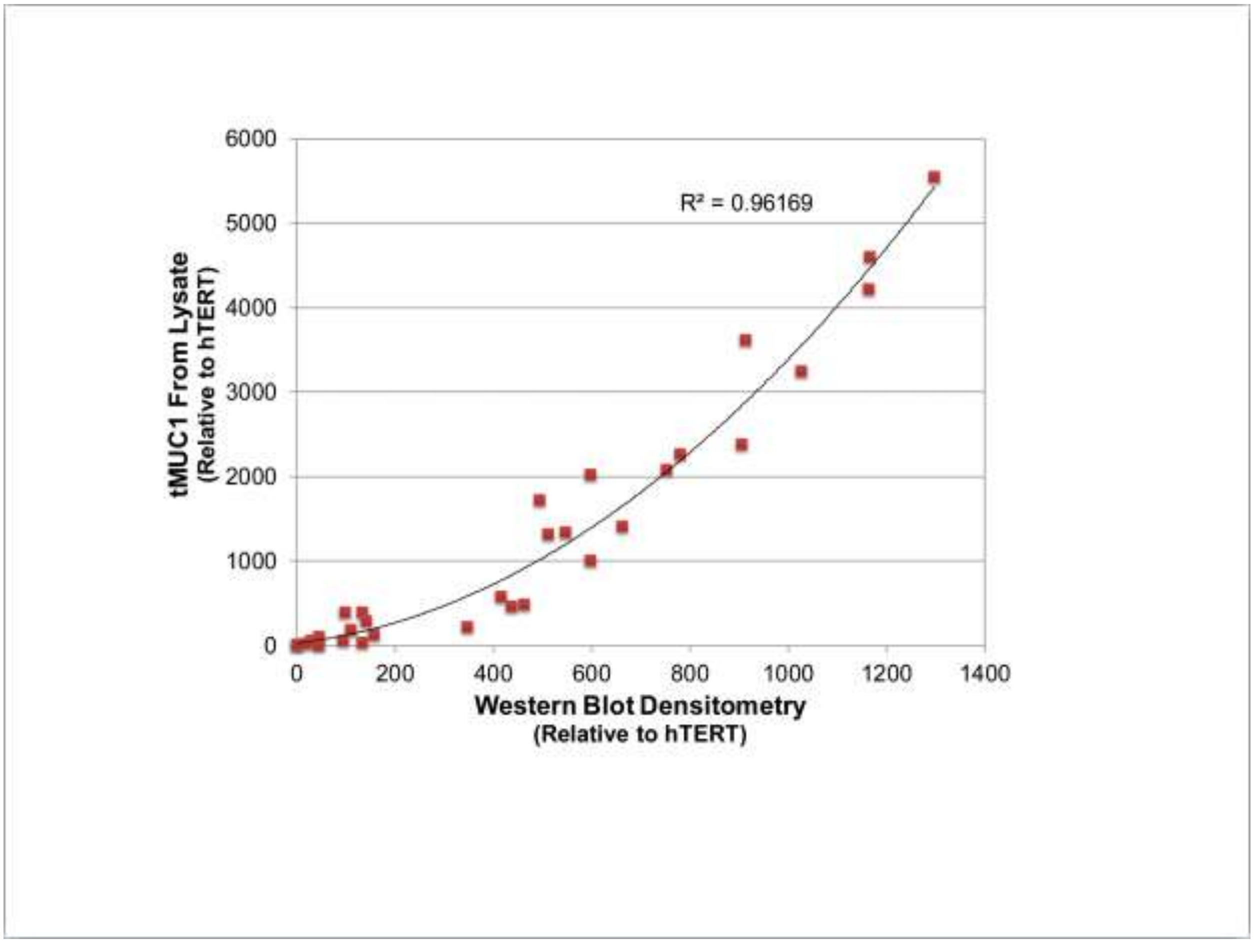

Supplementary Figure 2: Lysate tMUC1 correlation with western blot densitometry: tMUC1 concentration in breast cancer cell lysate correlates with western blot densitometry of breast cancer cell lines ($R^2 = 0.96$).
